# Anti-Leishmanial Activity (*In Vitro* and *In Vivo*) of Allicin and Allicin Cream Using *Leishmania major* (Sub-strain Zymowme LON4) and Balb/c Mice

**DOI:** 10.1371/journal.pone.0161296

**Published:** 2016-08-18

**Authors:** Dina M. Metwally, Ebtesam M. Al-Olayan, Manal F. El-Khadragy, Badriah Alkathiri

**Affiliations:** 1 Zoology Department, Faculty of Science, King Saud University, Riyadh, KSA; 2 Parasitology Department, Faculty of Veterinary Medicine, Zagazig University, Zagazig, Egypt; 3 Zoology & Entomology Department, Faculty of Science, Helwan University, Cairo, Egypt; National Centre For Cell Science, INDIA

## Abstract

**Background:**

*Leishmania* is a unicellular protozoan parasite that produces several human diseases, ranging from localized self-healing cutaneous lesions to deadly visceral infections.

**Objective:**

The effect of allicin on the growth of *Leishmania major (L*. *major)* promastigotes was evaluated under *in vitro* conditions. Moreover, the efficacy of a topical allicin cream was examined in BALB/c (Bagg albino, laboratory-bred strain of the House Mouse) mice with cutaneous leishmanial lesions compared to the currently used drug, sodiumstibogluconate (pentostam).

**Methods:**

Cytotoxiciy and promastigote proliferation were measured. Different concentrations (50, 100, 150, and 200 μM) of liquid allicin were tested on *L*. *major* promastigotes twice: after 24 and 48 hours using an MTT colorimetric assay. In the *in vivo* condition, the efficacies of allicin cream and liquid allicin at two concentrations (0.15 μM/mouse and 0.30 μM/mouse) were evaluated. Serum factors of the control and treated groups were tested to evaluate the toxic effects of allicin on the liver and kidney.

**Results:**

Allicin at a concentration of 50 μM inhibited the growth of *Leishmania* promastigotes. Topical application of allicin cream reduced lesion sizes in mice. No significant differences in biochemical analysis were observed between the control and treated groups.

**Conclusions:**

Allicin has antileishmanial effects under *in vitro* and *in vivo* conditions and may be used in clinical applications.

## Introduction

**Fig 1:**
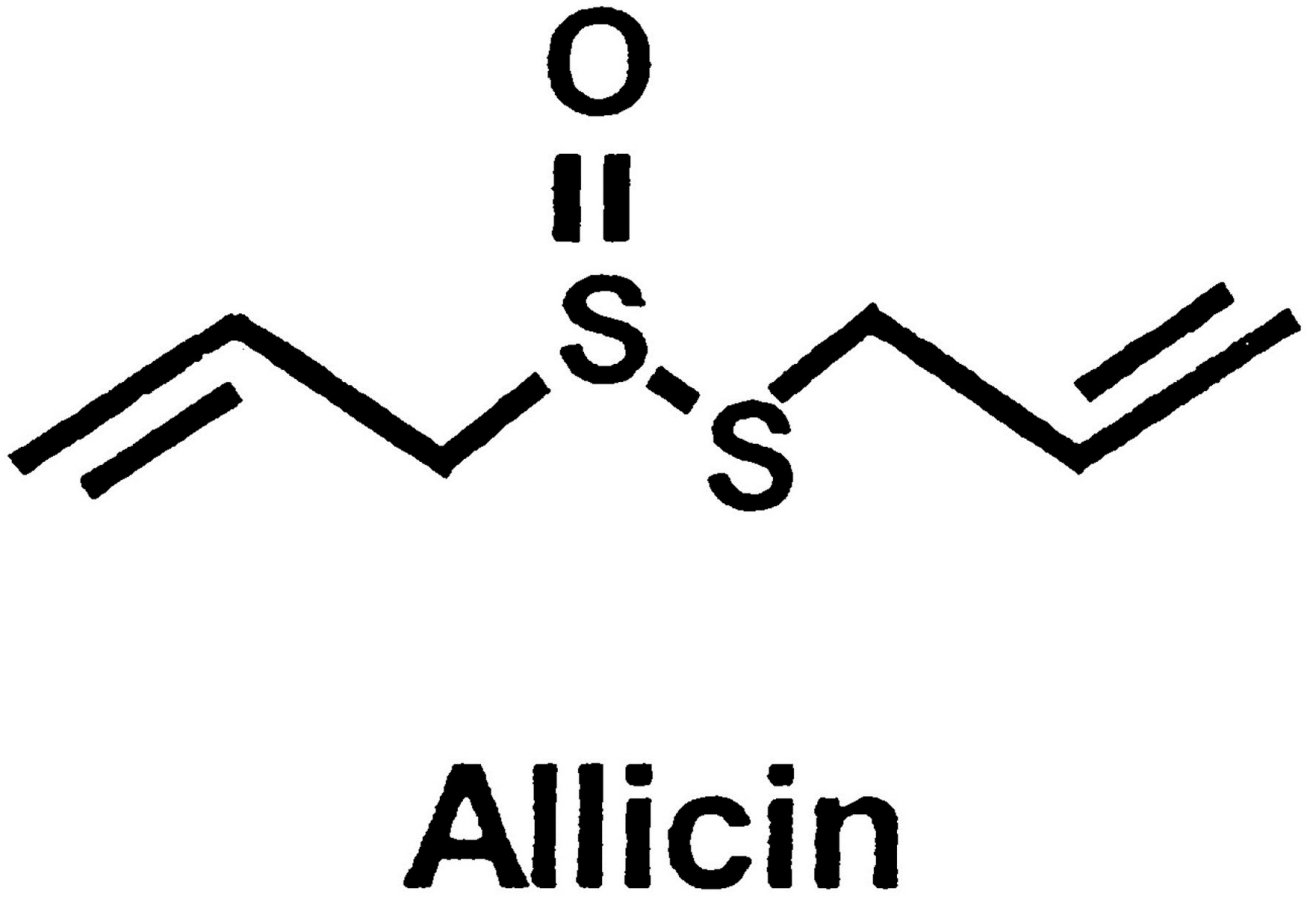
Chemical Composition of Allicin.

Leishmaniasis is a poverty-associated disease with several different forms, including Cutaneous Leishmaniasis (CL), Mucocutaneous Leishmaniasis (MCL), and Visceral Leishmaniasis (VL) [1]. Approximately 95% of CL cases occur in the Americas, the Mediterranean basin, the Middle East, and Central Asia. Over two-thirds of new CL cases occur in 6 countries: Afghanistan, Algeria, Brazil, Colombia, Iran, and the Syrian Arab Republic. An estimated 0.7 million to 1.3 million new cases occur worldwide annually [2]. CL has a broad spectrum of presentations, which typically include either self-healing or chronic lesions of the skin. The primary drug treatments for CL are antimony compounds (sodium stibogluconate or pentostam and meglumine antimoniate). However, these compounds have adverse effects, and drug resistance and relapse after treatment can occur [3–5]. Garlic (*Allium sativum*) is one of the oldest plants used as a medicine; it has been considered a valuable healing agent by many different cultures for thousands of years. Sulfur compounds of the plant, such as allicin, diallyl trisulphide, and ajoene, can reduce the development of different protozoan parasites, including *Giardia lamblia*, *Leishmania major*, *Leptomonas colosoma*, *Crithidia fasciculata*, *Cryptosporidium baileyi*, *Tetratrichomonas gallinarum*, *Histomonas meleagridis*, *Plasmodium berghei*, and *Trypanosoma* spp. [6,7]. Allicin (Fig 1) inhibits the proliferation of promastigotes of *Leishmania donovani* and *L*. *infantum in vitro* [8]. The aim of the present study was to evaluate antileishmanial activity of allicin under *in vitro* and *in vivo* conditions.

## Materials and Methods

### *L*. *major* isolate

*L*. *major* promastigotes (ZymowmeLON4) of a Saudi sub-strain were used and maintained with Roswell Park Memorial Institute (RPMI)-1640 medium supplemented with fetal bovine serum (FBS) (Sera Laboratories International, Horsted Keynes, UK), 100 U/mL penicillin + 100 mg/mL streptomycin (BioWhittaker, Verviers, Belgium), and 1% L-glutamine in 25-mL culture flasks. Each flask was incubated on its side in a standard incubator set at 25°C. This incubation method increases the medium aeration, allowing the cells to recover and grow faster [9].

### Source of allicin

Allicin was obtained in both liquid (1000 ppm) and cream (50 ml Allimed Cream) forms from Allicin International Ltd. (Rye, East Sussex, UK). The drugs were stored at 4°C and were retrieved only during use.

### Anti-promastigote assay using MTT (*in vitro* assay)

Different concentrations of liquid allicin (1000 ppm) (Allicin International Ltd., Rye, East Sussex, UK) or pentostam (GlaxoSmithKline, London, UK) in RPMI medium were prepared. Exponential-phase *L*. *major* promastigotes in cultured media (1.5 x10^6^/mL) were seeded into a 96-well plate and treated with the desired concentrations of the drugs (50–200 μM). Control wells were left without treatment. Blank wells contained only media. All experiments were performed in duplicate. Plates were incubated at 26°C in 5% CO_2_ for 48 h. A modified MTT colorimetric assay was conducted using 3-(4.5-dirmethyl-thiazol-2-yl)-2,5-diphenyltetrazolium bromide (Sigma-Aldrich, St. Louis, MO USA) for the detection of promastigote viability. MTT reagent (250 μg/mL) was added to each well, plates were incubated for 4 h at 26°C, and dimethylsulfoxide (DMSO) was added to dissolve the formazan crystals. The amount of cleaved tetrazolium salts to formazan, which directly correlates with the number of metabolically active cells in the culture, was quantified using an enzyme-linked immunosorbent assay (ELISA) reader at 540-nm absorbance [10]. Cell viability was calculated using the following equation:

$$V=\frac{AT}{AC\times \left(100\right)}$$ (1)

Cell viability (V)

Absorbance of treated sample (AT)

Absorbance of control cells (AC)

All values are means of duplicate wells. The 50% inhibitory concentration (IC_50_), i.e., the drug concentration that reduces the rate of cell growth by 50% (IC_50_), was calculated, and the results are displayed as the means and standard deviations.

### Experimental animals (*in vivo* assay)

Seventy 8-week-old female BALB/c mice were obtained from the Female Center for Scientific and Medical Colleges, Riyadh, Saudi Arabia. *L*. *major* infections were initiated by intramuscular (IM) injection of 0.1 ml RPMI-1640 medium (each ml of medium had 10^7^ promastigotes) per mouse. Parasitemia was assessed every other day by observing the appearance of lesions (2–6 weeks post-infection). Mortality was checked daily. The animals were kept in wire-bottomed cages in a room under standard conditions of illumination with a 12-hour light-dark cycle and at a temperature of 25 ± 1°C for one week until the beginning of treatment. Animals were provided with tap water and a balanced diet ad libitum. All experiments were performed in accordance with the requirements of the local animal ethics committee of the University of Salman bin Abdul-Aziz University (SAU) (IRB number:SAU-2014-Para-721/PI), including the joint work between the College of Science (King Saud University) and the Parasitology Department (Salman bin Abdul-Aziz University). The IRB approved of the animal research experiments. The Faculty of Medicine at Salman bin Abdul-Aziz University reviewed the protocol, assessing animal welfare and study design, and they approved of the present protocol involving animal research. No animals died during the course of the experiment, except by humane euthanasia. That is, no animals were euthanized prior to the end of the experiments, and no deaths occurred prior to the end of the experiments. Mice were sacrificed at the end of our experiment by decapitation according to the rules of the Animal Ethics Committee of our institution. The animals were divided into 7 groups, with 10 mice in each group. The seven groups were group I (negative control), group II (positive control), group III (infected and treated with pentostam [120 mg/kg I/M]), group IV (prophylactically treated with oral allicin [0.15 ml/mouse] once a day for two weeks before infection), group V (infected and treated with oral allicin [0.15 ml/mouse] once a day), group VI (infected and treated with oral allicin [0.3 ml/mouse] once a day), and group VII (infected and treated with allicin cream twice a day). Treatment was initiated when local lesions were apparent. The mice were treated daily for 4 continuous weeks. Each week, the lesion size was measured before and after treatment with vernier calipers in two diameters (a, b). The lesion size was calculated using the following formula:

$$\text{Lesion Size }\left(\text{LS}\right)=\frac{a+b}{2}$$ (2)

### Biochemical analysis

To assess the toxic effects of allicin in the livers and kidneys of the mice, serum samples were collected from the mice. Alanine aminotransferase, aspartate aminotransferase, urea, and creatinine were measured using commercial kits (Roche) with a Reflotron ^®^ Plussy system machine (Roche, Mannheim, Germany), and the results were statistically analyzed for assessment before and after treatment.

### Histopathological examination

Tissue samples were fixed in 10% neutral formalin for 24 hours, and paraffin blocks were obtained and routinely processed for light microscopy. Slices of 4–5 μM thickness were obtained from the prepared blocks and stained with hematoxylin-eosin. The preparations were visualized using a Nikon microscope at a magnification of ×400.

### DNA extraction

Total genomic DNA was extracted from each lesion using a Q-BIO gene kit (USA), and its concentration was determined using a NanoDrop. The DNA was then stored at –20°C until further use.

### PCR

Six DNA samples representing the different groups (1 pentostam, 1 allicin prophylaxis, 1 allicin [0.15 ml/mouse], 1 allicin cream, 1 control infected, and 1 control non-infected), were examined by Random Amplified Polymorphic DNA PCR (RAPD-PCR). Amplification was performed in a total volume of 25 μL, including 12.5 of μL GoTaq^®^GreenMaster Mix (Promega, Cat# M712c), 10.5 μL of RNAse-free water, 1 μL of DNA extraction, and 1 μL of primer (forward primer (5′-AGCTGGATCATTTTCCGATG-3′) and reverse primer (5′- ATCGCGACACGTTATGTGAG). The RAPD-PCR reaction was run as follows: 5 minutes at 95°C followed by 45 cycles of 1 minute at 94°C, 1 minute at 35°C and 1 minute at 72°C. In addition, we included another step of 10 minutes at 72°C and subsequent cooling to 4°C. The amplified DNA products were separated by electrophoresis on a 1.5% agarose gel. The DNA bands were stained with ethidium bromide (SIGMA, USA), visualized under UV light [11] and digitally photographed. RAPD band patterns of all 6 samples were compared with each other.

### Statistical analysis

Data are presented as the means and standard errors of the mean or standard deviations using the Statistical Package for the Social Sciences (SPSS v. 22, Chicago, IL, USA). The results are expressed as the means ± standard errors of the mean (SEM). All statistical comparisons between the control and the treated groups were performed using One-Way Analysis of Variance (ANOVA) followed by Dunnett’s post-hoc test for multiple comparisons. Significance was assigned at the level of (P<0.05). In the anti-promastigote assay using MTT, comparisons between groups were performed using Student’s t-test [12].

## Results

### Anti-promastigote effect (*in vitro* assay)

The means of the promastigote survival percentages of both allicin and pentostam were compared statistically using different concentrations of both agents (Table 1). According to the MTT cytotoxicity assay, the promastigotes were highly sensitive to allicin. The median lethal concentration of allicin (IC_50_) was 50 μM. At 50 μM, allicin showed statistically higher promastigote survival at 46.9%, relative to pentostam at 40.2% (p<0.01). Promastigote survival suddenly dropped to 15.1% at 100 μM allicin, a change that was significantly different from the 26.1% observed in pentostam (p<0.004). Using 150 μM allicin, promastigote survival reached 11.5%, while it was 25.5% with pentostam, a difference that was significant (p < 0.001). Promastigote survival dropped to 11.2% when using 200 μM allicin, compared to only 25% in the case of pentostam, and the difference between groups was significant (p < 0.001) (Table 1).

**Table 1:** Cytotoxic Effects of Different Concentrations of Allicin and Pentostam on the Multiplication of *L*. *Major* Promastigotes using an MTT Assay.

Dose	Group	N	Mean promastigotesurvival ± Standard deviation	Standarderror of themean	t	p-value
**0 μM**	pentostam	2	100 **±** 1	0.6		
	allicin	2	100 **±** 1	0.6		
**50 μM**	pentostam	2	40.20 **±** 1.5	0.9	- 4.5	0.01*
	allicin	2	46.9 **±** 2	1.2		
**100 μM**	pentostam	2	26.1 **±** 2.5	1.4	6.1	0.004*
	allicin	2	15.1 **±** 1.7	0.9		
**150 μM**	pentostam	2	25.5 **±** 2	1.1	8.9	0.001*
	allicin	2	11.5 **±** 1.7	1		
**200 μM**	pentostam	2	25 **±** 1.9	1	8.9	0.001*
	allicin	2	11.2 **±** 1.7	1		

Values are given as the means **±** SD

* Significant at p ≤ 0.05

### In vivo study

Clinically, cutaneous lesions in all infected groups started with redness and swelling at the site of inoculation on the 3^rd^ week of the infection. Swelling increased progressively, and crust formation occurred, with gangrene starting to develop by the 4^th^ week of infection. Lesion size was measured twice a week for the studied groups, and their mean was calculated (Table 2). In the infected control mice, the mean LS increased gradually to 9.8 ± 1.24 mm by the 4^th^ week of the experiment. It was observed that LS started to decrease gradually after the treatment regimen was initiated. The mean LS values were 6.50 ± 1 mm in the prophylactic group, 7.29 ± 1.36 mm in the pentostam-treated group, 6.55 ± 1.17 mm in the group treated with allicin (0.15 and 0.3 μM/mouse), and 4.67 ± 1.54 mm in the group treated with allicin cream. There was no significant difference in the mean LS values between the oral allicin groups and the pentostam group, whereas the mean difference between allicin cream and the control groups was significant (p < 0.05; Table 2).

**Table 2:** Lesion Size (mm) on Different days Post Infection and Treatment.

Different doses	1^st^ reading (mm	2^nd^ reading (mm)	p-value
	Mean±SD	Mean±SD	
**Control**	7.16±0.76	9.8±1.24	<0.0001
**Pentostam (120 mg/kg)**	7.4±1.95	7.29±1.36	0.876
**Prophylactic Allicin**	8.13±1.50	6.50±1.1	0.108
**Allicin (0.15 μM/mouse)**	8.15±1.55	6.55±1.17	0.109
**Allicin (0.3 μM/mouse)**	8.15±1.55	6.55±1.17	0.109
**Allicin cream**	7.35±1.97	4.67±1.54	0.039*

*Significant at p < 0.05

### Biochemical analysis

No significant differences were noted between the healthy control groups and the treated groups (p > 0.05; Table 3). This result revealed that the application of allicin had no aggressive effects on liver and kidney factors.

**Table 3:** Mean ± SD and Independent t-test for ALT, AST, Urea, and Creatinine for the Positive Control, Pentostam (120 mg/kg), Prophylactic Allicin, Allicin (0.15 mM/mouse), Allicin (0.3 mM/Mouse), and Allicin Cream Groups Compared to Healthy Controls.

Parameters	Group	N	Mean ± S.D.	% Change (compared to c-)	P value
**ALT (IU/L)**	Control negative	10	48.60 ± 1.94	100.00	
	Control positive	10	63.06 ± 2.30	129.76	0.001*
	Pentostam (120 mg/kg)	10	38.30 ± 4.51	78.81	0.001*
	Prophylactic allicin	10	45.80 ± 3.45	94.24	0.200
	Allicin (0.15 mM/mouse)	10	45.80 ± 3.45	94.24	0.200
	Allicin(0.3 mM/mouse)	10	45.80 ± 3.45	94.24	0.200
	Allicin cream	10	45.80 ± 3.45	94.24	0.200
**AST (IU/L)**	Control negative	10	48.60 ± 1.94	100.00	
	Control positive	10	63.06 ± 2.30	129.76	0.001*
	Pentostam (120 mg/kg)	10	46.60 ± 3.80	95.88	0.59
	Prophylactic allicin	10	46.50 ± 3.80	95.80	0.479
	Allicin (0.15 mM/mouse)	10	46.50 ± 3.80	95.80	0.479
	Allicin (0.3 mM/mouse)	10	46.50 ± 3.80	95.80	0.479
	Allicin cream	10	45.80 ± 3.45	94.24	0.200
**Urea (mg/dL)**	Control negative	10	29.60 ± 11.64	100	
	Control positive	10	30.00 ± 5.96	101.35	0.999
	Pentostam (120 mg/kg)	10	37.20 ± 13.80	125.68	0.218
	Prophylactic allicin	10	29.59 ± 6.00	100	1.00
	Allicin (0.15 mM/mouse)	10	37.18 ± 13.79	125.66	0.218
	Allicin(0.3 mM/mouse)	10	37.18 ± 13.79	125.66	0.218
	Allicin cream	10	30.02 ± 5.97	101.36	0.999
**Creatinine(mmol)**	Control negative	10	1.60 ± 0.52	100.00	
	Control positive	10	2.40 ± 0.52	150.00	0.066
	Pentostam (120 mg/kg)	10	2.20 ± 0.79	137.50	0.227
	Prophylactic allicin	10	2.40 ± 0.52	150.00	0.066
	Allicin (0.15 mM/mouse)	10	2.00 ± 1.15	125.00	0.570
	Allicin(0.3 mM/mouse)	10	2.00 ± 1.15	125.00	0.570
	Allicin cream	10	2.00 ± 1.15	125.00	0.570

*Significant difference between the studied groups compared to the healthy controls when p ≤ 0.05.

### Histopathological examination

**Fig 2:**
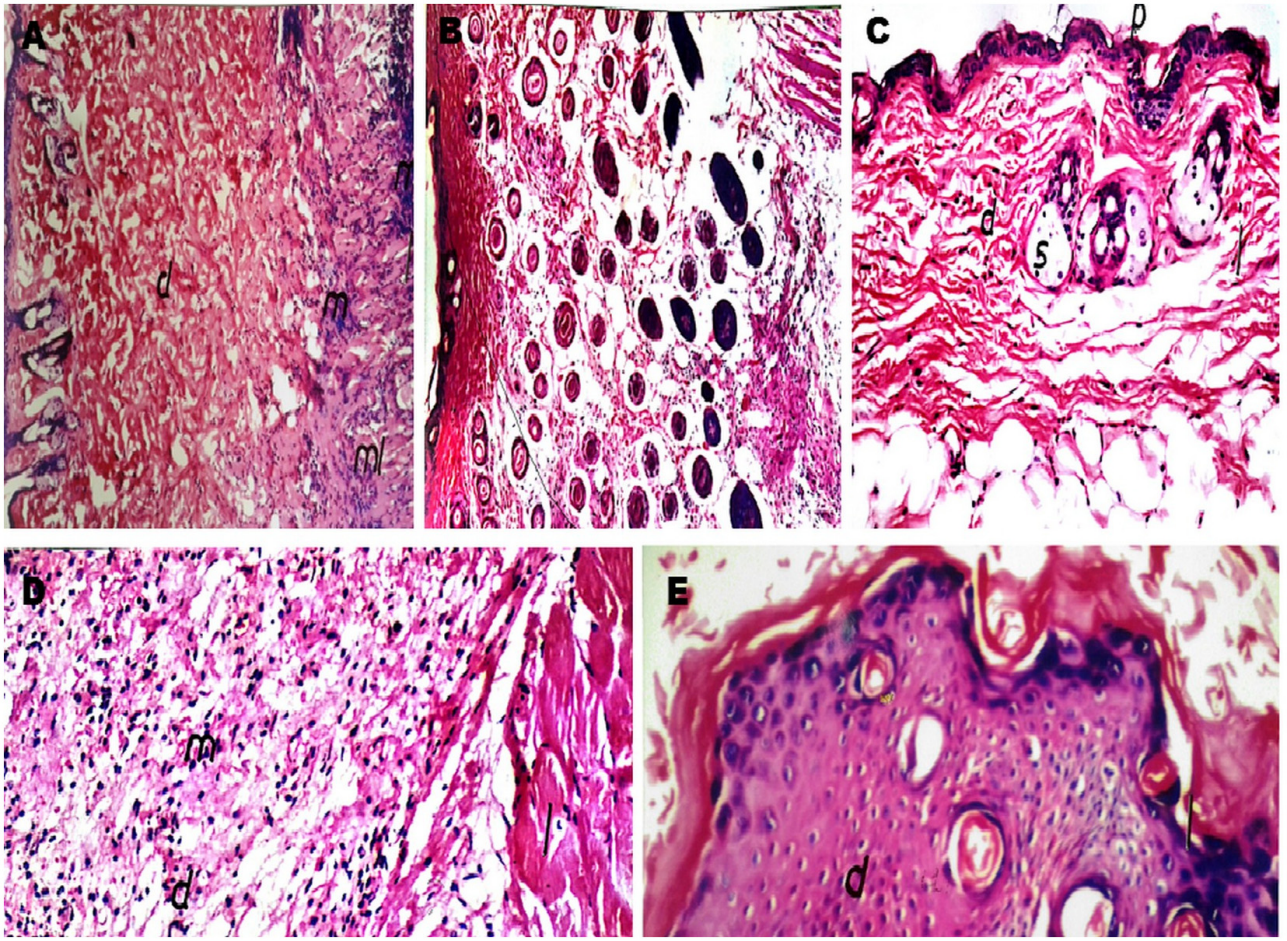
Light Microscopy of Mouse Skin. (A) Skin section of the positive control group at 4 weeks post-infection showing infiltration by a massive number of inflammatory cells in the subcutaneous and muscular tissue. (B) Pathological changes in mice treated orally with allicin at 4 weeks post-treatment showing edema with infiltration by moderate numbers of inflammatory cells in the subcutaneous tissue and musculature (m). (C) Allicin cream-treated group at the 4^th^ week post-treatment, showing an intact epidermis (p) and dermis (d) with remarkable reduction in the inflammatory response. (D) Prophylactic group showing few inflammatory cells in the underlying subcutaneous and adipose tissues. (E) Pentostam-treated mice at 4 weeks post-treatment showing hyperkeratosis and acanthosis in the epidermis (H & E staining, x400).

*L*. *major* produces a variety of cutaneous lesions, ranging from narrow cutaneous lesions, which settle spontaneously, to more severe mucocutaneous lesions (Fig 2A), depending on the host’s immune response. Regarding mice of the infected-treated group, the swelling increased progressively until the 3rd week of infection, when allicin and pentostam were applied. Swelling started to decrease gradually after the onset of the treatment regimen (Fig 2B, 2C, 2D and 2E). At the end of treatment (7th week of the infection), the skin appeared normal with no clinical relapse.

### PCR

**Fig 3:**
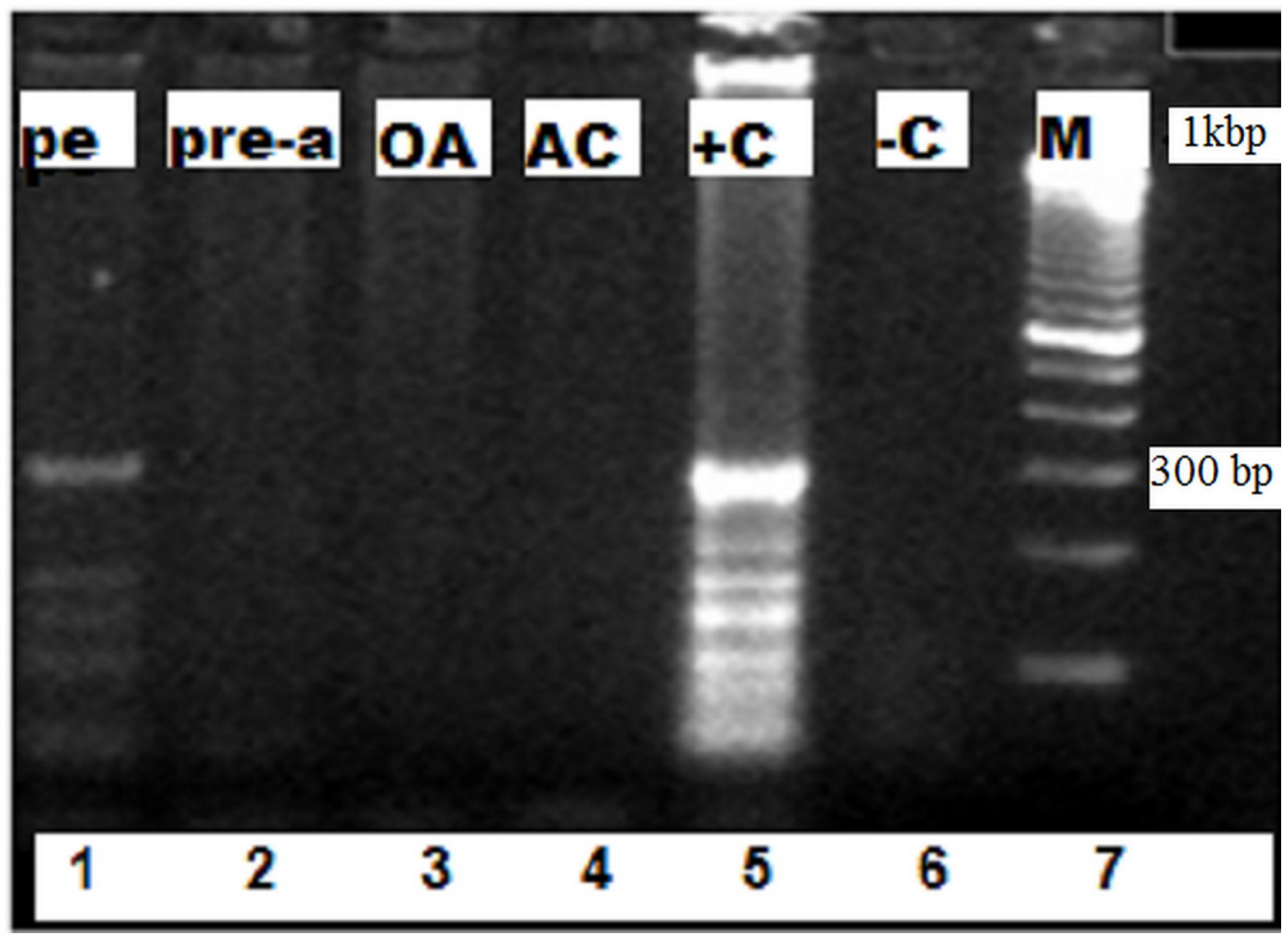
Agarose Gel (1.5%) Electrophoresis of PCR Amplification for the Identification of *L*. *Major*. M: 1000-bp DNA ladder marker, Lane 1: treatment with pentostam (Pe), Lane 2: prophylaxis with allicin (Pre-a). Lane 3: treatment with liquid oral allicin (OA). Lane 4: allicin cream (AC). Lane 5: *L*. *major* (+ C). Lane 6: negative control (- C).

As shown in (Fig 3), PCR-RFLP analysis provided further information about the effects of pentostam (lane 1) and other drugs, such as allicin (prophylactic: lane 2, oral: lane 3, and cream: lane 4), on the treatment of *L*. *major*. The RFLP pattern disappeared from samples of mice that were treated with pentostam and allicin compared to the 300-bp positive controls (lane 5).

## Discussion

The drugs currently used for leishmaniasis possess several limitations, such as high toxicity, management difficulties, and the development of resistance [13], increasing the need for safer and more effective drugs. Further studies on the treatment of leishmaniasis with natural and herbal elements [14] are needed. Allicin has dose- and time-dependent cytotoxic effects on *L*. *major* promastigotes. Promastigotes showed survival of 11.2%, compared to only 25% in the case of pentostam. The approximate IC_50_ was 50 μM.

The inhibition observed after 24 h of treatment was dramatic; however, pentostam displayed only mild inhibition in cell proliferation after 24 h compared to allicin. After 48 h of treatment, both allicin and pentostam showed very similar inhibitory effects, which continued for up to 72 h of treatment. Other studies have shown that the approximate IC_50_ values for allicin were 30 μg /mL [15] for promastigotes of *L*. *major* and 10–30 μM for promastigotes of both *L*. *donovani* and *L*. *infantum* [8]. The combination of liposomal amphotericin (AmB) and allicin ranged from being moderately synergic to synergic at low concentrations against both promastigotes (0.07 μM AmB plus 35.45 μM allicin induced 95% growth inhibition) and amastigotes (ca. 45% reduction with 0.05 μM AmB plus 10 μM allicin) [16].

The mechanism of action behind allicin’s effectiveness has been partially attributed to its rapid reaction with thiol groups [17,18], but the actual intracellular targets responsible for its cytostatic / cytocidal effects remain largely unknown. It has been reported that allicin induces apoptosis [19]. Topical application of allicin cream indicated that using this compound on a *L*. *major* lesion reduces LS. The mean LS was reduced to 4.67 mm after using allicin cream, and this reduction was statistically different from the control group (9.8 mm). The results revealed no significant differences between the treatment and control groups, and allicin had no negative effects on hepatic and renal factors. However, 120 mg/kg pentostam significantly decreased the concentrations of ALT serum levels. This result is in conflict with a previous study [20], in which pentostam-treated groups experienced acute and chronic kidney failure compared to a control group. In addition, our findings are not consistent with another study [21], in which pentostam caused inflammation of liver cells and impaired liver functions.

Pathological investigation revealed surface epithelial ulceration and localized dermal infiltration of specific inflammatory cells composed of macrophages mixed with few lymphocytes and neutrophils [11,12,22]. The aggregations of infected macrophages, granulocytes, and lymphocytes at the sites of intradermal inoculation appeared, and ulceration occurred consistent with previous findings [11,12,15]. The histological structure of the epidermal and dermal layers was normal at the 2^nd^ and 3^rd^ weeks of treatment. These findings were similar to those reported by other investigators [17,19]. Sections taken from the skin of mice treated with allicin cream showed an intact epidermis with remarkable reduction in inflammatory cells. Skin sections from the pentostam-treated group showed hyperkeratosis and acanthosis in the epidermis, along with inflammatory cell infiltration in the dermal and subcutaneous tissues. The diagnosis of leishmaniasis in mice is based on the appearance of ulcers on the skin and on an evaluation of the stage of amastigotes in clinical materials. Many studies cannot differentiate between *Leishmania* spp. due to their homogeneous morphologies [1,23]. Studies using PCR techniques to examine the antiparasitic activity of allicin against CL are scarce. Pentostam is effective in curing *L*. *major* in infected mice. The most effective treatment included intralesional injections, after which 85% of all treated mice in multiple experiments showed marked regression in the lesion size [24]. The use of pentostam or allicin could improve lesion healing and parasite resolution in BALB/c mice co-infected with *L*. *major*.

Collectively, the results of this study demonstrated for the first time that allicin displays an antileishmanial effect under *in vitro* and *in vivo* conditions. Various concentrations and application methods of allicin are required to further examine the effectiveness of allicin in the treatment and healing of human CL lesions. We recommend future molecular studies to further examine the apoptotic pathway of this molecule.

## Supporting Information

S1 File (PDF)

S2 File (PDF)
