# Supplementary material for: Anti-Leishmanial Activity (In Vitro and In Vivo) of Allicin and Allicin Cream Using Leishmania major (Sub-strain Zymowme LON4) and Balb/c Mice
Source: PLoS One. 2016 Aug 18;11(8):e0161296. doi: 10.1371/journal.pone.0161296 (PMC4990270; doi:10.1371/journal.pone.0161296)
Supplement: S1 File — (PDF) [file pone.0161296.s001.pdf]

A Guide to Your iThenticate® Report

The purpose of the iThenticate® report included in AJE's Publication Ready Editing service is to identify similarity between text in your manuscript and text in published sources, such as published journal articles. This similarity may be considered plagiarism by your target journal, preventing your manuscript from being reviewed and slowing your path to publication.

What does the iThenticate® report include?

1) **A highlighted copy of your manuscript**. The highlighted portions indicate text that is similar to published sources.

2) **A list of the specific published sources**. These sources are ranked by percentage similarity to your manuscript text.

3) **A similarity index**. The similarity index is the overall percentage similarity between the text of your manuscript and published text. To reduce the risk of false positives, we applied iThenticate® only to the main manuscript text and **not** to the bibliography or any quotations. Additionally, short portions of text (<10 words) flagged as similar to published sources have been excluded.

How should I use the iThenticate® report?

You should pay special attention to these issues:

- Large blocks of similar text, such as a whole sentence or a series of sentences within the same paragraph
- Repeated similarity to the same source(s)

The following issues may be less concerning:

- Similar text consisting of standard phrases that are commonly used in your field, rather than being specific to one or a few particular sources
- Similar text consisting of descriptions of methods that you previously published; note that some journals consider this acceptable, whereas others do not.

What should I do next?

Note that the criteria for text similarity vary from journal to journal but that many journals do use iThenticate® to determine whether a manuscript is eligible for review. If you notice large blocks of similar text or repeated similarity to the same source, consider rewriting the similar sections of text in your own words. Remember that if you do rewrite parts of your manuscript, you can submit the revised text for free re-editing and re-formatting.

Please note that a Chinese version of this document is available upon request (support@aje.com)
